# Robust Formation of an Epithelial Layer of Human Intestinal Organoids in a Polydimethylsiloxane-Based Gut-on-a-Chip Microdevice

**DOI:** 10.3389/fmedt.2020.00002

**Published:** 2020-08-07

**Authors:** Woojung Shin, Yoko M. Ambrosini, Yong Cheol Shin, Alexander Wu, Soyoun Min, Domin Koh, Sowon Park, Seung Kim, Hong Koh, Hyun Jung Kim

**Affiliations:** 1 Department of Biomedical Engineering, The University of Texas at Austin, Austin, TX, United States; 2 Severance Fecal Microbiota Transplantation Center, Severance Hospital, Department of Pediatrics, Yonsei University College of Medicine, Seoul, South Korea; 3 Department of Oncology, Dell Medical School, The University of Texas at Austin, Austin, TX, United States

**Keywords:** polydimethylsiloxane, hydrophobicity, surface functionalization, organoids, extracellular matrix, cell attachment, gut-on-a-chip

## Abstract

Polydimethylsiloxane (PDMS) is a silicone polymer that has been predominantly used in a human organ-on-a-chip microphysiological system. The hydrophobic surface of a microfluidic channel made of PDMS often results in poor adhesion of the extracellular matrix (ECM) as well as cell attachment. The surface modification by plasma or UV/ozone treatment in a PDMS-based device produces a hydrophilic surface that allows robust ECM coating and the reproducible attachment of human intestinal immortalized cell lines. However, these surface-activating methods have not been successful in forming a monolayer of the biopsy-derived primary organoid epithelium. Several existing protocols to grow human intestinal organoid cells in a PDMS microchannel are not always reproducibly operative due to the limited information. Here, we report an optimized methodology that enables robust and reproducible attachment of the intestinal organoid epithelium in a PDMS-based gut-on-a-chip. Among several reported protocols, we optimized a method by performing polyethyleneimine-based surface functionalization followed by the glutaraldehyde cross linking to activate the PDMS surface. Moreover, we discovered that the post-functionalization step contributes to provide uniform ECM deposition that allows to produce a robust attachment of the dissociated intestinal organoid epithelium in a PDMS-based microdevice. We envision that our optimized protocol may disseminate an enabling methodology to advance the integration of human organotypic cultures in a human organ-on-a-chip for patient-specific disease modeling.

## INTRODUCTION

Polydimethylsiloxane (PDMS) is silicone polymer that has been used in a variety of microfluidic devices, including the human organ-on-a-chip microphysiological systems (MPS) (1). PDMS has provided great applicability in biological experiments with its high biocompatibility, adjustable elastomeric property, low-cost expense, high gas permeability, and optical transparency (2–4). The elastance of PDMS has been particularly appreciated to emulate intestinal peristalsis-like motions in intestinal MPS such as the gut-on-a-chip (5–8) by applying cyclic rhythmical stretching deformations. However, the hydrophobic surface of PDMS that causes poor protein adhesion and cell attachment remains a critical challenge in PDMS-based microfluidic devices regardless of its compelling stability and implementability. To improve the surface hydrophobicity, PDMS devices are first activated with high energy, using oxygen plasma (9, 10), corona beam (11, 12), or UV/ozone (13, 14) treatment methods. In some cases, chemical-based functionalization has been applied to expose functional groups (i.e., amine group, aldehyde group) that have a high affinity to extracellular matrix (ECM) proteins via covalent bonding (15, 16). On the activated PDMS surface, ECM proteins are coated to enhance the cell attachment. Many studies have demonstrated various methods for protein immobilization on a PDMS surface and the culture of immortalized human intestinal epithelial lines or primary human intestinal epithelium (Table 1).

There has been a major paradigm shift in biomedical research utilizing the three-dimensional (3D) intestinal organoid-derived epithelium to better reflect patients’ genetic susceptibility or variants and patient-specific phenotypes (28). Organoids from primary stem cells retain the diverse epithelial populations and disease-specific traits (29), which is a great promise to build a patient-oriented customizable model toward Precision Medicine (30). However, a critical limitation of the 3D organoid system is that the organoid body prevents access to the luminal interface for studying the epithelial interactions with exogenous antigens such as microorganisms, drugs, nutrients, and toxins because the lumen is enclosed inside the spherical organoid. While a microinjection of experimental materials (i.e., living bacterial cells or chemical compounds) into the lumen of an organoid has been applied to introduce those antigens into the lumen side of an organoid, this technique requires a microinjector and labor-intensive skill. More importantly, an invasive process compromises the physical epithelial barrier, by which an undesirable leakage or a barrier disruption may happen. In addition, the growth of 3D organoids embedded inside a hydrogel considerably limits the application of necessary biomechanics such as fluid shear stress or mechanical deformations mimicking intestinal peristalsis (5). Furthermore, this static and closed culture condition seriously limits stable maintenance of a living population of the gut microbiome over long-term co-cultures with the organoid epithelial cells (31, 32). Thus, the recreation of a polarized epithelial interface of 3D organoids with accessible apical–basolateral ports is a critical component for manipulating the intestinal cellular microenvironment that contains microbial, mesenchymal, and immune elements under mechanically active movements (33, 34).

However, it has been challenging to grow an organoid-derived epithelium on a PDMS-based microfluidic device for a couple of reasons. First, primary organoid-derived epithelial cells often rapidly lose viability over the dissociation process due to the dissociation-induced apoptosis (35). Second, a defined methodology for surface activation is required on the PDMS microchannel when the dissociated organoid cells are applied for the cultures compared to the immortalized intestinal cell lines. Indeed, it has been observed that the commercially available intestinal cell lines are robust enough to adhere, after a simple pretreatment of the hydrophobic PDMS surface (5, 7, 8, 36), compared to the organoid-derived epithelial cells (Table 1). While some reports demonstrated the microfluidic cultures of the intestinal organoid-derived epithelium (17–23), it has been elusive to define a reliable and reproducible protocol for robust surface activation of a PDMS microchannel because of the limited quantitative information from the published works. Thus, a systematic optimization of the method that allows a robust surface activation, stable ECM coating, and strong attachment of the organoid-derived intestinal epithelium is an unmet need for holistic applications to various PDMS-based MPS models that deploy organoid-derived cells.

In this study, we report an optimized method for generating an intact epithelial layer of human colonoid-derived cells in a PDMS-based gut-on-a-chip. We tested a couple of reported methods for the activation and functionalization of a PDMS surface. We also validated the impact of a post-functionalization process that affects the qualified ECM coating as well as the attachment of a dissociated organoid-derived epithelium. Finally, we demonstrated the reproducible formation of an organoid-derived epithelial monolayer in the PDMS-based microfluidic gut-on-a-chip.

## METHODS

### Organoid and Cell Line Culture

The de-identified human colonoid lines derived from three normal donors (C103, CN136, and HJK026) or two patients with Crohn’s disease (CD; CD7517 and CD8) were randomly used in this study. We further annotated “CD8” into “iCD8” and “nCD8” to indicate the intestinal organoid lines derived from the inflamed and non-inflamed region from a CD patient, respectively. We obtained each line from various institutions as follows: CD5717 (The University of Chicago), HJK026 (Dell Medical School at UT Austin; IRB-2017–06-0114), iCD8 and nCD8 (Yonsei University College of Medicine; IRB-4–2017-0223), and C103 and CN136 (Baylor College of Medicine). In terms of the race/ethnicity background of each donor, colonoid lines denoted as the CD7517, HJK026, CD8, C103, and CN136 were derived from a White female, White male, an Asian male, a Black female, and a White female, respectively.

Human primary colonoids were isolated from colonic biopsy as previously described (29). Briefly, colonoids embedded in 30 μL of Matrigel (Corning) were cultured with 500 μL of the complete medium in a 24-well plate (Corning) in a humidified CO_2_ incubator at 37°C. The basal medium was prepared by mixing 10 mM 2-[4-(2-hydroxyethyl)piperazin-1-yl]ethanesulfonic acid (HEPES; Gibco), 1× GlutaMAX (Invitrogen), 100 units/mL penicillin (Thermo Fisher Scientific), and 100 μg/mL streptomycin (Thermo Fisher Scientific) in Advanced Dulbecco’s Modified Eagle Medium (DMEM)/F12 (Gibco). The conditioned medium was prepared by culturing Wnt3a-producing L cells (ATCC, CRL 2647), R-spondin1 (Rspo1) cells (Trevigen), and Noggin-secreting HEK293 cells (Baylor College of Medicine). To prepare a complete medium, we mixed basal, Wnt, Rspo1, and Noggin media at 10/75/10/5% (v/v). Murine recombinant epidermal growth factor (EGF) (50 ng/mL; PeproTech), SB202190 (30 μM; Sigma-Aldrich), A-8301 (500 nM; Sigma-Aldrich), Gastrin (10 nM; Sigma Aldrich), *N*-acetylcysteine (1 mM; MP Biomedicals), nicotinamide (10 mM; Sigma-Aldrich), N2 (1×; Gibco), and B27 (1×; Gibco) were also added to the complete medium. The complete medium was changed every other day to support organoid growth. The fully grown organoids were passed once a week. When organoids were passed, 500 μL Cell Recovery Solution (Corning) was treated per well, and the plate was incubated at 4°C for 30 min. After harvesting the organoids in a 15-mL conical tube, organoids were pelleted by centrifugation at 100× *g* at 4°C for 5 min, then 1 mL of TrypLE Express (Gibco) was added per tube, and the tube was incubated at 37°C for 2 min. After deactivating the enzymatic activity by adding 4 mL of 10% (v/v) fetal bovine serum (FBS, Gibco)-containing Advanced DMEM/F12 followed by pipetting with a P1000 micropipette for at least 20 times, fragmented organoids were pelleted (100× *g*, 4°C, 5 min), resuspended with fresh Matrigel on ice, and then plated in each well of a 24-well plate. The viability of dissociated organoid cells was assessed by using trypan blue (final concentration, 0.2%, w/v).

The HT-29 human colorectal adenocarcinoma line was obtained from American Type Culture Collection (ATCC HTB38). SW480 (human colorectal adenocarcinoma line) and HTC116 (human colorectal carcinoma line) were obtained from the Harvard Digestive Disease Center. The culture medium was prepared by mixing DMEM (Gibco) containing 4.5 g/L D-glucose, 25 mM HEPES, 20% (v/v) FBS, 100 units/mL penicillin, and 100 μg/mL streptomycin. These cells were cultured in a T-75 flask (Celltreat Scientific) until cells were confluent, then 1 mL Trypsin-EDTA solution (0.25%, Fisher Scientific) was treated for 5 min at 37°C after rinsing with Ca^2+^ and Mg^2+^-free phosphate-buffered saline (PBS, Gibco) twice. After dissociation, cells were collected in a 15-mL tube, spun down (300× *g*, 4°C, 5 min), resuspended with a fresh medium (final cell density, 5 × 10^6^ cells/mL), then introduced into a gut-on-a-chip device that underwent UV/ozone surface activation.

### Modification of the PDMS Surface for Testing Organoid Cell Attachment

The PDMS slabs were prepared by casting 15 mL of degassed PDMS prepolymer (elastomer:curing agent = 15:1 (w/w); Sylgard 184, Dow Corning) in a petri dish (Corning) followed by a curing step at 60°C for 6 h. Cured PDMS slabs (∼2 mm thick) were cut into a square (10 × 10 mm), then placed on a 24-well plate in aseptic condition. The PDMS slabs were sterilized by dropping 70% (v/v) ethanol for 2 min, rinsed three times with deionized water, and dried at 80°C overnight. For the (3-aminopropyl)triethoxysilane (APTES; Sigma-Aldrich) treatment, PDMS slabs were exposed to UV/ozone (Jelight Company Inc.) for 1 h and treated with 2% (v/v) APTES solution diluted in pure ethanol (200 proof, Pharmco) at room temperature for 1 h. After washing with 100% ethanol, the PDMS slabs were completely dried at 80°C overnight. For the polyethyleneimine (PEI)/glutaraldehyde (GA) treatment, PDMS slabs were exposed to oxygen plasma (Femto Science Inc.; atmospheric gas was used, target pressure at 5 × 10^−1^ torr, power at 125 W) for 1 min and treated with 1% (v/v) PEI solution (Sigma-Aldrich) diluted in deionized water at room temperature for 10 min followed by 0.1% (v/v) GA solution (Electron Microscopic Sciences) in deionized water at room temperature for 20 min. The PEI/GA-treated PDMS slabs were then washed with sterilized water twice and coated with ECM proteins. Control PDMS slabs were prepared by exposure to UV/ozone for 1 h, followed by coating with ECM proteins without any additional surface treatment. Prior to seeding the dissociated colonoid cells, the control and APTES- and PEI/GA-treated PDMS slabs were coated with 1% (v/v) Matrigel (Corning) and 90 μg/mL of collagen I (Fisher Scientific) in the organoid basal medium at 37°C for 2 h and washed with PBS.

The colonoids were harvested after 7 days of culture by adding the Cell Recovery Solution at 4°C for 30 min, transferred in a 15-mL tube, and centrifuged (100× *g*, 4°C, 5 min). The colonoid pellet was incubated with 1 mL TrypLE Express solution (Gibco) at 37°C for 10 min, followed by centrifugation at 100 × *g* for 5 min. The organoid fragments resuspended in a complete medium were further dissociated by repeated pipetting, then filtered with a cell strainer (cutoff size, 300 μm; Corning) to obtain a single-cell suspension (final density, ∼1 × 10^4^ cells/mL). An aliquot (100 μL) of the dissociated suspension was seeded onto each PDMS slab, then incubated in a humidified 5% CO_2_ incubator at 37°C for 3 h. Next, a complete organoid medium (1 mL) was gently added to each well and further incubated overnight. After washing the cells with Ca^2+^ and Mg^2+^-free PBS to remove unbound dead cells or cell debris, cell morphology was monitored using a phase-contrast microscope (DMi1, Leica). To quantify initial cell attachment, the cell surface area was measured from manually segmented cells using the freehand selection tool of ImageJ software (version 1.52p).

### Fabrication and Surface Treatment of a Gut-on-a-Chip

A gut-on-a-chip device was made using a soft lithography method (5, 37). Briefly, the upper and lower microchannel layers of a gut-on-a-chip were prepared from cured PDMS (elastomer:curing agent = 15:1, w/w). The upper and lower microchannels have dimensions of 1 × 10 × 0.5 mm and 1 × 10 × 0.2 mm (width × length × height), respectively. A porous PDMS membrane (pore size, 10 μm in diameter) compartmentalized the upper and lower microchannels. Each layer was bonded to a porous membrane by corona treatment (BD-20A corona treater, Electro-Technic Products) followed by incubation at 60°C overnight. Each microchannel was connected to silicone tubing (Tygon 3350, ID 1/32′′, OD 3/32′′, Beaverton) with a connector (a blunt-end needle, 18 G; Kimble Chase) to introduce the surface treatment reagents, washing solution, and culture medium into the microchannels. To activate the surface of microchannels in a gut-on-a-chip, an assembled gut-on-a-chip device was fully dried at 60°C for 12 h, exposed to UV/ozone for 1 h, then treated with 1% (v/v) PEI, diluted in deionized water, at room temperature for 10 min, followed by incubation with 0.1% (v/v) GA, and diluted in deionized water, at room temperature for 20 min. After washing the microchannels with sterilized water, the device was completely dried at 60°C overnight. The device was cooled down at room temperature for 10 min before the ECM coating.

### Effects of Washing Condition on the Residual ECM in a Gut-on-a-Chip

To evaluate the effect of the washing steps on the ECM-coated microchannel of a PDMS-based gut-on-a-chip, we prepared a sterilized gut-on-a-chip exposed to UV/ozone for 1 h, then treated with either PEI/GA or APTES for the modification of the PDMS surface as previously described. After the treatment of PEI/GA or APTES in the microfluidic channel of a gut-on-a-chip, microchannels were washed with 100% ethanol, followed by drying at 80°C overnight. The microchannels in a gut-on-a-hip were coated with an ECM mixture of 1% (v/v) Matrigel and 90 μg/mL collagen I in a serum-free basal medium at 37°C for 2 h, then washed with an ECM-free basal medium at different flow rates of 30 and 300 μL/h for 1 h using a syringe pump (BS-8000 infusion pump, Braintree Scientific). For the manual washing, microchannels in a gut-on-a-chip were washed by manually infusing 200 μL of the basal medium for 1 min (mean flow rate, ∼12,000 μL/h, corresponding shear stress in the upper microchannel, ∼0.752 dyne/cm^2^). The area of residual ECM was estimated using the color threshold tool of ImageJ (hue: 0–255, saturation: 0–255, brightness: 115–255 for laminin and 110–255 for collagen).

### Microscopic Imaging

The ECM proteins adhered on the surface of a microchannel in a gut-on-a-chip were fixed with 4% (w/v) paraformaldehyde (PFA; Electron Microscopy Science) at room temperature for 15 min, blocked with 2% (w/v) bovine serum albumin (BSA; Sigma Aldrich) solution, then washed with PBS. The microchannel was incubated with 0.1% (v/v, diluted in 2% BSA solution) mouse anti-collagen I monoclonal antibody (Abcam) and 2.5 μg/mL rabbit anti-laminin polyclonal antibody (Abcam) at 4°C overnight. Next, we introduced the secondary antibodies of Alexa Fluor 488-conjugated goat polyclonal anti-mouse IgG (Abcam) and Alexa Fluor 555-conjugated goat polyclonal anti-rabbit IgG (Abcam) at room temperature under light protection for 1 h. The expression of ZO-1 tight junction protein was visualized by fixing (4% PFA, room temperature, 15 min), permeabilizing (0.3% Triton X-100, room temperature, 30 min; Ricca Chemicals), and blocking (2% BSA solution, room temperature, 1 h) the colonoid-derived monolayer followed by sequential incubation with the primary (10 μg/mL of rabbit anti-ZO-1 polyclonal antibody, room temperature, 3 h; Invitrogen) and secondary antibodies (Alexa Fluor 555-conjugated goat polyclonal anti-rabbit IgG, light protected, 3 h; Abcam). After washing with PBS, nucleus staining was performed using 4′,6-diamidino-2-phenylindole dihydrochloride (DAPI; 5 μg/mL; Thermo Scientific). Imaging analysis was performed using a laser-scanning confocal microscope (DMi8, Leica). The cell occupancy was quantified by normalizing the area that a cell monolayer covered in the microchannel by the entire surface area of the microchannel. Acquired images were processed using either LAS X (Leica) or ImageJ (version 1.52p).

### Statistical Analysis

All the quantitative data were expressed as the mean ± standard error (SEM). A one-way analysis of variance (ANOVA) followed by a Bonferroni test was applied in Figures 2B, 3D,E and Figures S2D,E. Two-tailed unpaired *t*-test was performed in Figure 5C. Differences between groups were considered statistically significant when *P* < 0.05.

## RESULTS

### Functionalization of the PDMS Microchannel

**FIGURE 1 |:**
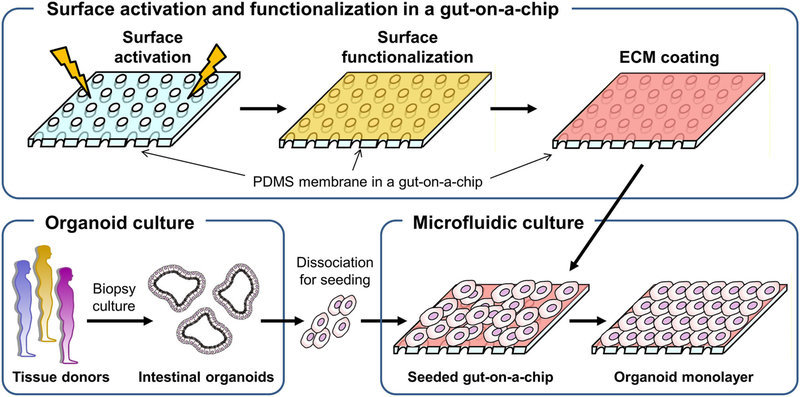
A schematic of the experimental procedure to generate a monolayer of the intestinal organoid epithelium in a gut-on-a-chip microfluidic device. A porous membrane embedded in a gut-on-a-chip was demonstrated in the upper panel, in which plasma or UV/ozone treatment is applied to activate the surface of the PDMS membrane. On the surface-activated PDMS membrane, chemical functionalization followed by ECM coating was performed. The biopsy-derived intestinal organoids obtained from tissue donors are enzymatically and mechanically dissociated, then introduced into the microchannel that is preactivated and chemically modified in the gut-on-a-chip to form an organoid epithelial monolayer.

To culture intestinal organoids in a gut-on-a-chip made of PDMS, the surface of PDMS needs to be manipulated to decrease the hydrophobicity and increase the adhesion of ECM proteins (Figure 1). A common sequence of the experiment is the surface activation (i.e., oxygen plasma or UV/ozone treatment), chemical-based surface functionalization, ECM coating, and seeding of dissociated organoid cells. Various methods that have been applied for the manipulation of the PDMS surface are summarized in Table 1. Notably, human intestinal cell lines including Caco-2 (5–8, 36), HT-29 (Figure S1A; goblet-like human colorectal adenocarcinoma), SW480 (Figure S1B; human colorectal adenocarcinoma), and HCT116 (Figure S1C; human colorectal carcinoma) can robustly attach to the PDMS surface preactivated with UV/ozone treatment followed by ECM coating (1% Matrigel and 30 μg/mL collagen I) for 1 h. It is also noted that chemical functionalization is not necessary for the attachment of human intestinal immortalized cell lines, suggesting that the attachment of those cell lines is robust and reproducible with minimal group-to-group variations. However, it has been considerably challenging to adhere the dissociated primary organoid-derived epithelium on the PDMS surface when the aforementioned surface treatment protocol for primary cell lines was applied without modification (Table 1).

**FIGURE 2 |:**
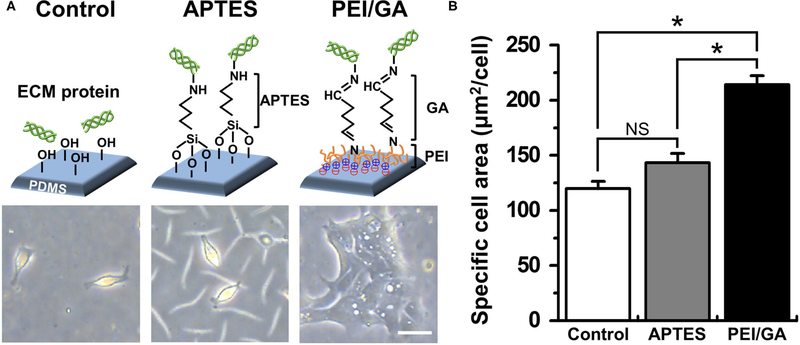
Optimization of the functionalization of a PDMS surface. **(A)** The schematic in the upper panel displays the experimental design and the mode of surface functionalization. The lower panel shows the phase-contrast micrographs of the morphology of dissociated organoid epithelium after 24 h since seeding (cell density, ~1 × 10^4^ cells/mL). **(B)** Quantification of the mean area of attached cells normalized by the total number of cells attached. We used normal colonoid (C103 line). In the Control group, UV/ozone treatment was applied. *P < 0.05. NS, not significant. *N =* 150. Bar, 20 μm.

Based on this motivation, we used a normal human colonoid line (C103) to optimize the attachment condition of the dissociated organoid epithelium on the PDMS surface. First, we selected two representative protocols using APTES (38, 39) or PEI/GA (16, 40) which have been applied to chemically modify the hydrophobic surface of PDMS. In this study, we used PDMS slabs to preliminarily evaluate which chemical shows better performance to induce rapid cell attachment on the PDMS surface pretreated with UV and ozone. We found that the PEI/GA group showed the best cell attachment, in which spread cell morphology strongly supported the uniform and stable attachment on the functionalized PDMS surface (Figure 2A). To quantitate the effectiveness of the surface modification on the attachment of the individual organoid epithelium, we measured the specific cell area defined as the mean area of individual single cells. The specific cell area of the PEI/GA group (213.98 ± 8.27 μm^2^/cell) was significantly (*P* < 0.0001) larger than both the Control (119.85 ± 6.55 μm^2^/cell) and APTES groups (143.25 ± 8.46 μm^2^/cell) (Figure 2B), suggesting that the PEI/GA group showed the best efficiency in cell attachment. The functionalization with APTES did not show significant difference from the control.

### Effect of Flow on the Removal of ECM in a Microchannel

**FIGURE 3 |:**
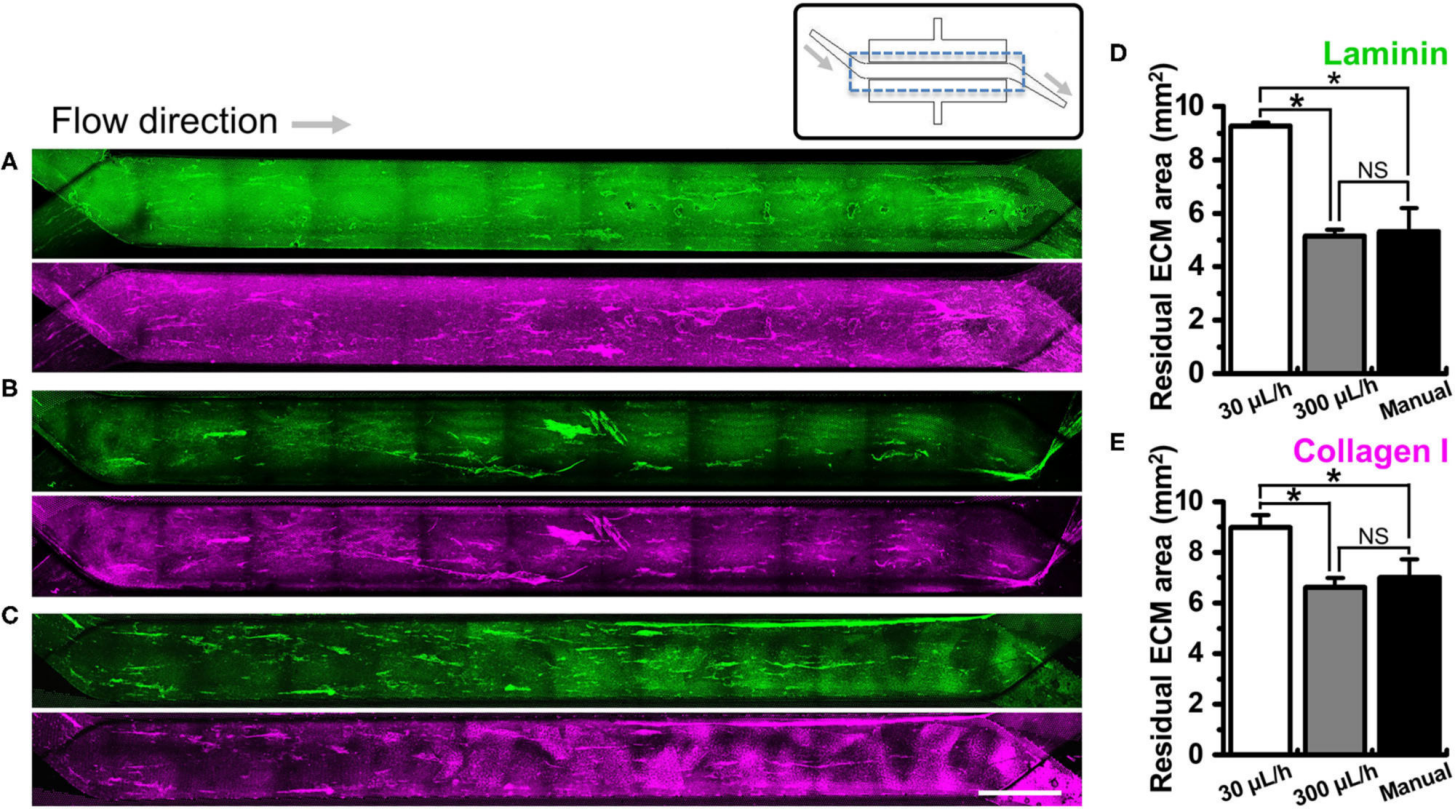
Effect ofthe flow on the removal of ECM in a microchannel of a gut-on-a-chip device. Different flow rates (i.e., 30, 300, and ~12,000 μL/h) were employed on the UV/ozone-treated, PEI/GA-functionalized PDMS microchannel followed by the coating with an ECM mixture [1 % (v/v) Matrigel and 90 μg/mL collagen I] for 2 h. The residual laminin (green) and collagen I (magenta) in the microchannel were visualized after washing at **(A)** 30 μL/h for 1 h, **(B)** 300 μL/h for 1 h, and **(C)** ~12,000 μL/h (i.e., a manual washing) for 1 min. The stitched fluorescence images were provided to show the efficiency of ECM coating along the entire microchannels of the gut-on-a-chip devices. A schematic in the upper panel displays the structure of a gut-on-a-chip, where a dotted box indicates the region of stitched images. Quantification of the residual mean area of laminin **(D)** and collagen I **(E)** was performed after the washing step. *P < 0.05. NS, not significant. *N =* 3. Bar, 1 mm.

We investigated if the washing step after ECM coating may perturb the adherent ECM on the PDMS microchannel of a gut-on-a-chip. To test this hypothesis, we performed UV/ozone treatment in the gut-on-a-chip followed by PEI/GA functionalization and ECM coating as previously described. Next, we let the ECM-free basal medium flow at various flow rates ranging from the conventional culture condition (i.e., 30 μL/h) up to manual flushing (∼12,000 μL/h, equivalent to the fluid shear stress at ∼0.752 dyne/cm^2^ in the upper microchannel). The quantification of the residual ECM was performed by visualizing laminin, the most abundant ECM protein in Matrigel (41), and collagen I. We found that both laminin and collagen I remained uniformly across the entire cell culture channel in the PEI/GA-functionalized gut-on-a-chip at 30 μL/h (Figure 3A; 9.27 ± 0.13 and 8.99 ± 0.48 mm^2^ for laminin and collagen I, respectively). On the contrary, a higher flow rate at 300 μL/h (Figure 3B) and ∼12,000 μL/h (Figure 3C) resulted in a decreased residual ECM area for both laminin (Figure 3D; 5.14 ± 0.25 and 5.31 ± 0.89 mm^2^ for 300 μL/h and ∼12,000 μL/h, respectively) and collagen I (Figure 3E, 6.61 ± 0.37 and 7.01 ± 0.71 mm^2^ for 300 μL/h and ∼12,000 μL/h, respectively). Interestingly, when APTES was applied for surface functionalization instead of PEI/GA, even the lowest washing flow rate at 30 μL/h resulted in the reduced residual ECM area (Figure S2; 5.80 ± 0.42 and 5.94 ± 0.81 mm^2^ for both laminin and collagen I, respectively). When the higher flow rate at 300 μL/h was applied, we observed that most of laminin and collagen I were removed (residual ECM area, 1.14 and 2.27 mm^2^ of laminin and collagen I, respectively) from the porous membrane. Manual washing also caused a significantly decreased area of the residual laminin (Figure S2D), whereas the residual area of collagen I was not affected by the manual washing condition (Figure S2E).

### Optimization of the Surface Functionalization With PEI/GA

**FIGURE 4 |:**
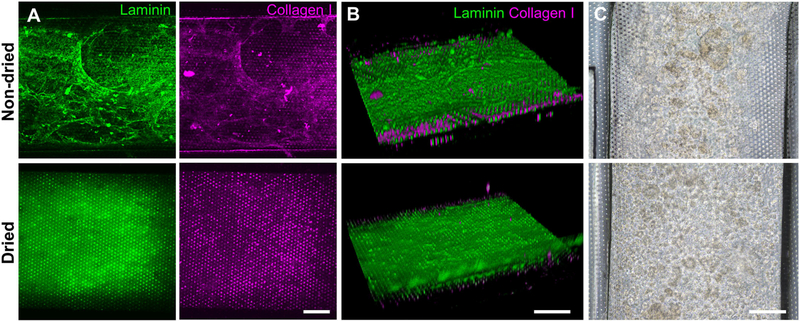
Effects of the drying step after the PEI/GA surface functionalization in a gut-on-a-chip. The post-functionalization without (top) or with (bottom) overnight drying was performed to evaluate the efficiency of ECM coating. Representative images of IF staining against laminin (green) and collagen I (magenta) with **(A)** a top-down view and **(B)** an angled view. **(C)** Phase-contrast images display the biased or uniform distribution of the organoid cells (CD, iCD8 line) seeded into the microchannel without (top) or with drying optimization process (bottom), respectively, in a gut-on-a-chip. Bars, 200 μm.

Based on the screening assessment of the surface functionalization (Figures 2, 3 and Figure S2), we further optimized the post-functionalization step to lead to a uniform distribution and attachment of the dissociated organoid cells. After the surface functionalization via PEI/GA treatment, we applied sterilized deionized water manually into the microchannels for washing. In our preliminary study, however, we observed that the ECM mixture that we applied after this washing step underwent a substantial aggregation of both laminin and collagen I inside the 500 μm-high microchannel, forming a web-like structure (Figure 4A, “Non-dried”). A 3D reconstruction of Z-stacked immunofluorescence (IF) images also revealed that the non-dried chip shows a 3D topology of a web-like ECM structure (Figure 4B, “Non-dried”). More importantly, the gelation due to the GA residue physically blocked a uniform distribution of dissociated organoid cells on the PDMS porous membrane (Figure 4C, “Non-dried”). The physical hindrance of cell seeding was not resolved even after the aggressive multiple manual flushing of the medium. Furthermore, organoid cells seeded into the upper microchannel were floated in the middle of the microchannel instead of the attachment on the surface of the coated PDMS porous membrane (Video S1), which seriously hampered to form a monolayer of the organoid epithelium.

**FIGURE 5 |:**
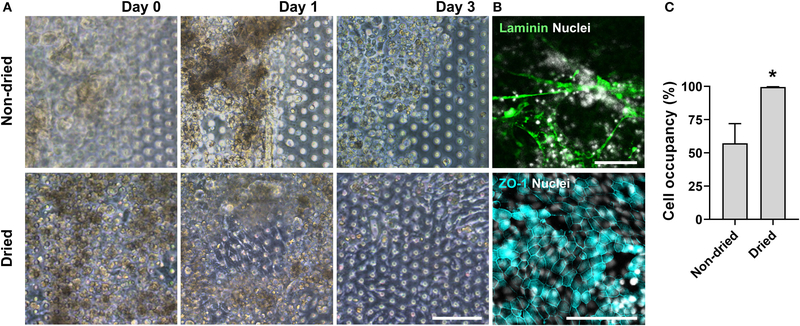
An optimized formation of an intestinal organoid monolayer in the gut-on-a-chip. **(A)** Phase-contrast micrographs showing cell morphologies (CD, iCD8 line) at different time points since the seeding. When the chips are not dried post-functionalization (“Non-dried”), dissociated organoid cells were not evenly distributed and thus could not form a monolayer. However, dried chip post-functionalization (“Dried”) let the dissociated organoid epithelium attach within 3h and established a full monolayer in 2–3 days. **(B)** Assessment of the monolayer formation of the dissociated organoid epithelium (Normal, C103 line) by staining laminin and nuclei (top) or tight junction protein ZO-1 (bottom). Without the post-functionalization drying step, cells adhered to the laminin web-like structure, whereas the drying step enabled cells to form an intact monolayer. **(C)** Quantitative measurement of the cell occupancy in the microchannels. The organoid lines that we applied in this chart include CN136 (normal), HJK026 (normal), iCD8 (CD), nCD8 (CD), and CD7517 (CD). *P < 0.05. *N =* 5. Bars, 100 μm.

**FIGURE 6 |:**
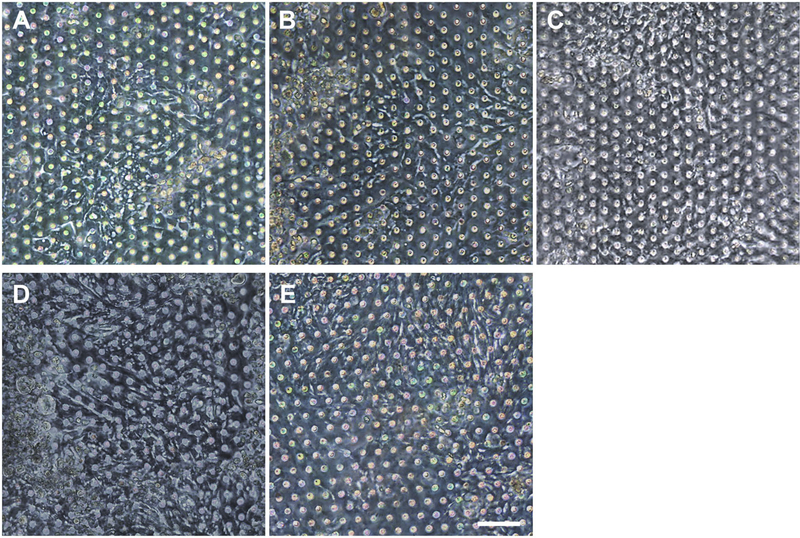
Verification of the reproducible formation of an organoid epithelial monolayer established using various human intestinal organoid lines. Using the optimized protocol, an epithelial monolayer derived from both normal and CD organoid lines was robustly and reproducibly established in a gut-on-a-chip. Phase-contrast micrographs disclose the monolayers derived from the human intestinal organoid lines of **(A)** C103, **(B)** CN136, **(C)** HJK026, **(D)** CD7517, and **(E)** nCD8 on day 3 of the culture. Bar, 100 μm.

We postulated that the residual GA in the microchannel may cause this undesirable 3D ECM structure. Therefore, we attempted to remove the residual liquid component in the microchannels by incubating the device in a dry oven at 60°C overnight, by which we completely removed the remaining GA at any corner of the microchannel. We found that this drying step significantly resolved the problem, where a uniform deposition of the coated ECM was observed without any 3D web-like gelation inside the microchannel in both 2D (Figure 4A, “Dried”) and 3D reconstruction imaging (Figure 4B, “Dried”). The dried chip achieved a uniform seeding of the dissociated organoid epithelium without biased seeding or cell hanging in the microchannel (Figure 4C, “Dried”). This consistent and even cell seeding consequently resulted in a robust cell attachment and monolayer formation. When the post-functionalization (e.g., drying step) was not performed (Figure 5A, “Non-dried”), cells seeded into the upper microchannel neither show a uniform distribution at Day 0 nor form a monolayer with adherent morphology on the coated PDMS porous membrane at Day 1 or 3. On the contrary, the optimized surface activation, functionalization, and post-functionalization conditions successfully led to the robust formation of a monolayer of the human intestinal organoid-derived epithelium in a PDMS-based gut-on-a-chip. The IF imaging analysis further confirmed that organoid cells used in the “Non-dried” condition did not achieve a monolayer and cells stuck on the randomly deposited laminin proteins (Figure 5B, “Non-dried”). On the contrary, a continuous formation of the tight junctional protein, ZO-1, was observed throughout the surface of a gut-on-a-chip, demonstrating the presence of an adequate epithelial monolayer with junctional integrity (Figure 5B, “Dried”). Quantitative assessment of the monolayer formation indicated the necessity of the drying step, where the “Dried” condition achieved 99.4% of the cell occupancy on Day 3 and the “Non-dried” condition showed 57.3% of the cell occupancy (Figure 5C). We verified that the monolayer formation was robustly achieved with all of the organoid lines we utilized in the study (Figure 6), where the cell–cell junctions (white contour between cells) were clearly observed via phase-contrast microscopy.

### Optimization of Other Experimental Factors

To achieve an overall optimized protocol to culture an intestinal organoid monolayer in a PDMS-based gut-on-a-chip, we also carried out optimization of other experimental steps (Table 2). In terms of the preparation of ECM mixture, three different ratios of collagen I and Matrigel mixture were prepared and verified for the assessment of cell attachment on the PDMS-based microchannel. Although the concentrations of collagen I and Matrigel were adjusted in a range of 30–120 μg/mL and 1–4% (v/v), respectively, all three conditions resulted in a successful cell attachment. Thus, we determined an optimized ratio of a mixture of ECM at 30 μg/mL collagen I and 1% (v/v) Matrigel. In terms of organoid dissociation, we optimized the treatment time of a commercially available dissociating reagent (here, TrypLE Express) for 5, 10, and 15 min. We found that the cell viability assessed by the trypan blue staining method shows higher than 85% in all groups without statistical significance. However, dissociation for 5 min was not sufficient to dissociate the whole organoids into single cells. Although the treatment of TrypLE Express for 10 vs. 15 min showed a similar degree of dissociation, dissociation for 10 min showed a better yield in forming a monolayer compared to the dissociated cells for 15 min. Thus, we confirmed an optimized dissociation time for 10 min using TrypLE Express solution. We also determined an optimized filtration step with a 300-μm strainer to remove insufficiently dissociated organoid cells or Matrigel debris. A smaller-sized strainer (e.g., 40 μm cutoff) did not effectively collect the dissociated organoid epithelium. To disseminate our organized protocol, we summarized all the optimization criteria and the final optimized conditions in Table 2.

## DISCUSSION

We report an optimized method for the generation of an epithelial monolayer derived from human intestinal organoids in a human gut-on-a-chip made of PDMS. To increase the efficiency of cell attachment on the PDMS surface in a microchannel, chemical-based surface functionalization was applied and optimized. We determined that the PEI/GA treatment followed by an overnight drying process achieved the most successful ECM coating and the attachment of a dissociated human primary organoid epithelium. We tested normal as well as Crohn’s disease patients’ organoid cells to maximize the implementability of our optimized protocol in other PDMS-based microdevices. Finally, we holistically summarized an overall optimized protocol in a table to disseminate our experimental methodology.

Although there have been a couple of studies to demonstrate a microfluidic culture of organoid-derived cells; currently there is no “gold standard” method to generate an epithelial layer of human intestinal organoids on the PDMS surface (Table 1). It is noted that the culture of primary intestinal organoids in a PDMS-based device is technically nascent. Available protocols are not always robust and reproducible because critical information for the experimental validation is often missing. For instance, some studies do not provide the detailed information of the plasma treatment (e.g., gas configuration, preincubation time under vacuum, and target pressure inside the plasma chamber). The critical information regarding the organoid dissociation and manipulation in a PDMS-based microfluidic device was not always sufficiently described in the previous reports, which considerably limits to disseminate an implementable protocol. Since the use of patient-derived organoids and their integration into the human organ-on-a-chip MPS have been rapidly emerging, it is important to share and validate different methodologies at various contexts of microphysiological systems (e.g., organ-specific adaptability, host–microbiome co-culture, inter-organ interactions). In our study, we provide a robust, reproducible, and implementable protocol for the readers to simply follow and establish a stable monolayer of the intestinal organoid-derived epithelium on a hydrophobic PDMS surface. To ensure the repeatability and reproducibility, we tested 6 different lines (three normal and three diseased) of human intestinal organoids in the study. Importantly, we confirmed that the different organoid lines did not show a significant difference in forming the epithelial monolayer in the PDMS-based device, suggesting that the reproducibility in culture yield using our optimized protocols was successfully verified. We also optimized the dissociation condition by accurately assessing the efficiency of cell attachment and the formation of a monolayer. A longer treatment of dissociation reagent may not be encouraging as the yield of monolayer formation was low, possibly due to the dissociation-induced apoptosis which ultimately failed cells to attach on the ECM coated PDMS surface (35).

PDMS has provided great applicability in biological experiments to build human intestine MPS models such as the gut-on-a-chip (5–8). However, one of the major challenging properties of PDMS is a hydrophobic surface that causes poor protein adhesion and cell attachment. While PDMS has been well applied for the culture of immortalized intestinal cell lines such as Caco-2 (5, 36), HT-29 (6), SW480, and HCT116, the cell attachment on the hydrophobic PDMS surface is particularly important when stem cells are being implemented in the MPS systems. Indeed, culturing human stem cells (both embryonic and adult) or stem cell-containing tissue biopsies are not trivial compared to the cultures of immortalized intestinal cell lines due to a limited knowledge on the manipulation of those primary cells in the PDMS-based microfluidic devices (42). Therefore, the establishment of a robust method to culture the organoid-derived epithelium on PDMS surfaces may expedite the progress and broaden the utility of organoid-based organ-on-a-chip studies.

Among various surface-manipulating agents (43, 44), PEI has shown a phenomenal outcome in altering the hydrophobic PDMS surface into the protein-friendly, hydrophilic environment (16, 45). It is noted that PEI binds to the PDMS surface by nonspecific electrostatic interactions, where its secondary and tertiary amine groups can provide an anchoring site for further improving the efficiency of surface functionalization (46). In addition to PEI treatment, GA can contribute to cross-link the amines in collagen and other ECM proteins (43, 47–49). In particular, GA can form strong covalent linkages between the ECM proteins and the PEI-modified PDMS surface, which results in a stable ECM protein immobilization. On the other hand, three ethoxy groups of APTES specifically bind with the hydroxyl groups of the activated PDMS surface. In contrast to the non-specific binding of PEI and PDMS surfaces, the ethoxy groups of APTES remaining unreacted may cause the less efficient outcome of the PDMS surface functionalization (50, 51). Hence, we postulate that the PEI/GA surface functionalization method could achieve a stable and uniform coating of ECM proteins on the PDMS microchannel than the APTES treatment. However, it has been reported that PEI and GA show cytotoxicity to fibroblasts or airway epithelium (52, 53); thus, a careful modification and optimization is necessary for the broader applications in the organoid–organ chip integrations. In the current study, we advanced this caveat by removing the residual PEI and GA in the microchannels. We validated that the incubation of the device in a dry oven at 60°C overnight to remove the residual PEI/GA. This approach provided not only the uniform seeding of dissociated organoid cells but also the non-toxic microenvironment for the culture of dissociated organoid cells in a PDMS-based device.

Regarding the washing effect on the ECM coating on the PDMS chip, higher flow shear in both 300 μL/h and manual flushing does not significantly reduce the attached ECM on the PDMS surface when the PEI/GA was used as a surface modifier compared to APTES. On the surface-modified PDMS with APTES, collagen I remained more abundant than laminin under the manual washing condition. It is supposed that the total amounts of collagen and laminin proteins in the ECM solution were different. The concentration of collagen in the ECM mixture (∼90 μg/mL) was approximately 1.5 times higher than that of laminin (∼48–72 μg/mL) (54). Also, the collagen may robustly bind with the activated PDMS surface via covalent bonding with abundant amine groups (55, 56). In contrast, cross-linkable functional groups of laminin are mainly located on its coiled region (57), which may result in a limited covalent bonding with the activated PDMS surfaces than collagen.

Finally, our optimized protocol can be applied to multiple different lines of human intestinal organoids. The validation of our optimized methodology using normal as well as patient organoids proposes a possible implementability of our protocol to other types of intestinal organoids such as the organoids from different regions of the intestine (e.g., small intestine vs. colon), different diseases such as colorectal cancer and ulcerative colitis, or different sources of the provider not only from human but also from other species such as canine (58), mouse (28), or pig (59). Since the demand for various MPS models using various silicone-based materials has progressively increased, we anticipate that our optimized protocol will potentially disseminate versatile usability and roust reproducibility in patient-derived disease modeling using an organ-on-a-chip device.

## Supplementary Material

SI

SI video

## Figures and Tables

**TABLE 1 |:** A summary of protocols for the functionalization and seeding of human intestinal organoid cells or immortalized intestinal cell lines in PDMS-based microfluidic devices.

Optimization factors	Intestinal organoids						Intestinal cell lines		
Surface activation	Oxygen plasma	N/A	N/A	Oxygen plasma	Oxygen plasma (2 min)	N/A	UV-Ozone (40 min)	Oxygen plasma (30 s)	N/A
Surface functionalization	2% APTMS (v/v, 30 min) Drying at 80°C, overnight	ER-1 and ER-2 solutions (Emulate, Inc.)	Sulfo-SANPAH^a^ (0.5 mg/mL)	2% APTMS (v/v, 30 min) Drying at 80°C, overnight	N/A	N/A	NA	N/A	N/A
ECM coating	Collagen I (200 μg/mL) + Matrigel (1 %, v/v) Incubated at 37°C for 2 h	Collagen I (200 μg/mL) + Matrigel (1%, v/v) dilution in PBS	Collagen I (200 μg/mL) + Matrigel (1%, v/v) dilution in PBS	Collagen IV (200 μg/mL in PBS) Incubated 37°C for 2 h	Matrigel (83 μg/mL) (Time not included)	Matrigel (2%, v/v) Incubated overnight at 4°C	Collagen (30 μg/mL) + Matrigel (1%, v/v)	Poly-L-lysine (0.01%) Fibronectin (2.5 μg/mL) Laminin (2.5 μg/mL) Collagen (0.01%) Fibronectin (50 μg/mL)	Fibronectin (50 μg/ml) Collagen (5 mg/mL), dried overnight Laminin (10 μg/mL) for 2 h
Cell source	Small intestinal organoids	Colonic organoids	Colonic organoids	Small intestinal organoids	iPSC-derived intestinal organoids	Colonic organoids	Caco-2 HT-29 SW480 HCT116	Caco-2	Caco-2
Cell dissociation	TrypLE (Time/concentration not mentioned)	TrypLE diluted in PBS (1:1 ratio; 2 min)	TrypLE diluted in PBS (1:1 ratio; 1 min 45 sec)	TrypLE (time/concentration not mentioned)	TrypLE (15–30 min)	0.25% Trypsin-EDTA at 37°C for 20 min	Trypsin/EDTA	Trypsin/EDTA	Trypsin/EDTA
Cell attachment	Overnight	Overnight	Overnight	Overnight	3–6 h	N/A	1 h	Overnight	N/A
References	(17–19)	(20)	(21)	(22)	(23)	(24)		(25, 26)	(27)
							Figure S1 & (5).		

N/A, not applicable due to the missing description in the manuscript.

a Sulfo-SANPAH: sulfosuccinimidyl 6-(4′-azido-2′-nitrophenylamino)hexanoate.

**TABLE 2 |:** A summary of the optimized protocol for creating an organoid-derived epithelial monolayer in a PDMS-based gut-on-a-chip device.

Sequence	Variables	Conditions	Optimized conditions
1. Surface activation	Activation method	• Plasma treatment • UV/ozone treatment	UV/ozone treatment
2. Surface functionalization	Type of chemical	• No functionalization • APTES • PEI/GA	PEI/GA
3. Post-functionalization	Wet vs. dry	• Wet • Drying at 60°C overnight	Drying at 60°C overnight
4. ECM coating	ECM concentration	• Collagen I (30 μg/mL) + Matrigel 1% (v/v) • Collagen I (60 μg/mL) + Matrigel 2% (v/v) • Collagen I (120 μg/mL) + Matrigel 4% (v/v)	Collagen I (30 μg/mL) + Matrigel 1% (v/v)
5. Cell dissociation	Treatment time of TrypLE Express	• 5 min • 10 min • 15 min	10 min
6. Filtration	Strainer cutoff size	• No filtration • 40-μm cell strainer • 300-μm cell strainer	300 μm cell strainer
7. Incubation for cell attachment	Incubation time	• 3 h • Overnight	Overnight

## Data Availability

All datasets presented in this study are included in the article/Supplementary Material.
