# Supplementary material for: Robust Formation of an Epithelial Layer of Human Intestinal Organoids in a Polydimethylsiloxane-Based Gut-on-a-Chip Microdevice
Source: Front Med Technol. 2020 Aug 7;2:2. doi: 10.3389/fmedt.2020.00002 (PMC7849371; doi:10.3389/fmedt.2020.00002)
Supplement: Supplementary file 1 [file Data_Sheet_1.PDF]

*Supplementary Material*

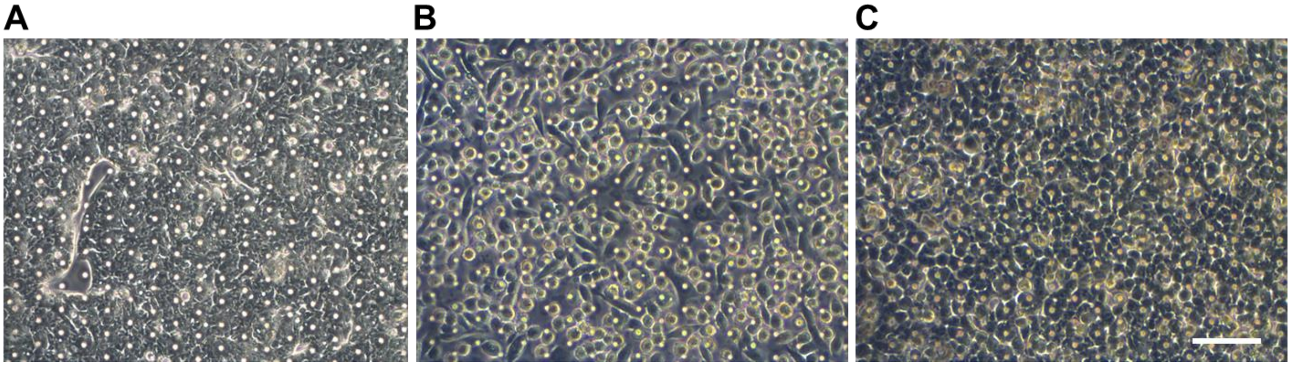


**Figure S1.** Attachment of various intestinal cell lines on the PDMS porous membrane in a gut-on-a-chip device. Intestinal cell lines that we tested include (A) HT-29, (B) SW480, and (C) HCT116. Cells resuspended in a culture medium at $5\times10^6$ cell/mL were seeded in a gut-on-a-chip pre-activated by UV/ozone treatment for 40 min followed by the coating with 1% (v/v) Matrigel and 30 µg/mL collagen I for 1 h. Phase contrast micrographs reveal that the each cell line can robustly attach on the surface of a PDMS porous membrane in a gut-on-a-chip pre-activated by conventional UV/ozone treatment. Bar, 100 µm.

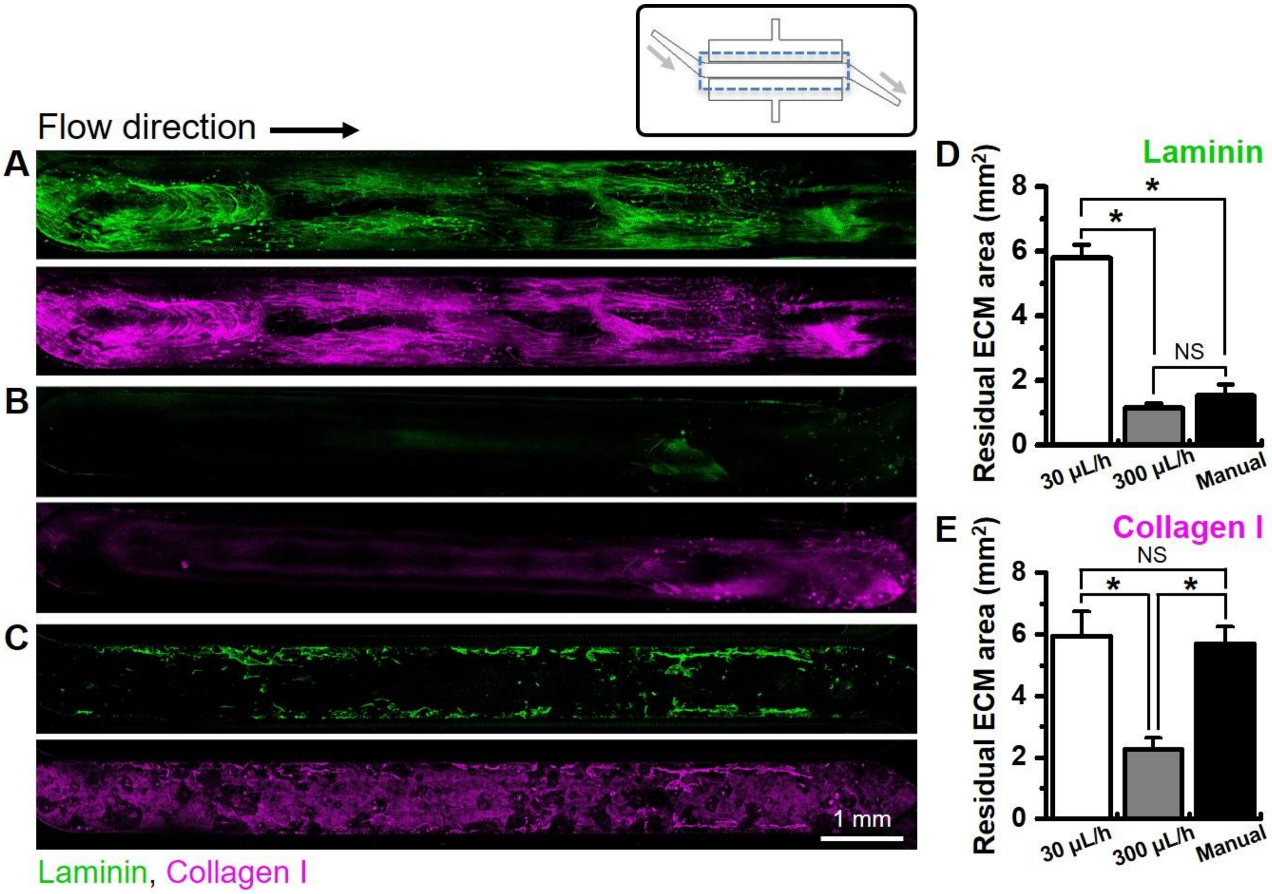


**Figure S2.** Profile of the residual ECM in response to the fluid flow in a microchannel pre-treated with APTES. After coating with a mixture of Matrigel (1%, v/v) and collagen I (90 µg/mL), ECM-free basal medium was perfused at (A) 30, (B) 300, and (C) ~12,000 µL/h (i.e., manual washing) for 1 h (at both 30 and 300 µL/h) or 1 min (at ~12,000 µL/h), respectively. The residual laminin (green) and collagen I (magenta) on the PDMS porous membrane are visualized via confocal microscopy. A schematic in the upper panel displays the structure of a gut-on-a-chip, where a dotted box indicates the region of stitched images. Quantification of residual (D) laminin and (D) collagen I was performed by measuring the residual ECM area using an image analysis software, Image J. *$P<0.05$. NS, not significant. N=3.

**Video S1.** A z-stacked movie displays the floating organoid cells observed at different focal planes in the microchannel of a gut-on-a-chip when the drying step in the post-functionalization was omitted. Dissociated organoids (normal organoid, CN136 line) seeded and cultured in a gut-on-a-chip at 30 µL/h for 24 h did not form a monolayer in the microchannel due to the web-like ECM scaffold. Organoid-derived cells were observed at different z-positions in the upper channel, where the focus in the video moves from the surface of the porous membrane to the ceiling of the upper microchannel.
